# The association between functional status and physical pain with depressive symptoms after a stroke event: A cross-sectional analysis of the China Health and Retirement Longitudinal Study 2018

**DOI:** 10.3389/fpsyt.2022.927856

**Published:** 2022-09-12

**Authors:** William Yang Zhao, Luwen Zhang, Yingfeng Wan, Xiaoying Chen, Yinzi Jin, Lin Zhang, Grace Sum, Ameera Katar, Lili Song, Craig S. Anderson

**Affiliations:** ^1^The George Institute for Global Health, Beijing, China; ^2^The George Institute for Global Health, University of New South Wales, Sydney, NSW, Australia; ^3^School of Health Services Management, Southern Medical University, Guangzhou, China; ^4^Department of Neurology, University of California, Davis, Davis, CA, United States; ^5^Department of Global Health, School of Public Health, Peking University, Beijing, China; ^6^Institute for Global Health and Development, Peking University, Beijing, China; ^7^School of Population Medicine and Public Health, Chinese Academy of Medical Sciences and Peking Union Medical College, Beijing, China; ^8^Melbourne School of Population and Global Health, The University of Melbourne, Melbourne, VIC, Australia; ^9^Saw Swee Hock School of Public Health, National University of Singapore, Singapore, Singapore; ^10^Department of Neurology, Royal Prince Alfred Hospital, Sydney, NSW, Australia

**Keywords:** stroke complications, disability, pain, depression, China

## Abstract

**Background:**

Stroke is a major cause of mortality and long-term physical and cognitive impairment. This study aims to: (1) examine the prevalence of depressive symptoms, disability and pain among Chinese adults with stroke; (2) test the associations of functional limitations and body pain with occurrence of depressive symptoms; (3) investigate gender and urban-rural disparities in these associations.

**Methods:**

This study utilized the data from the China Health and Retirement Longitudinal Study in 2018, involving 969 patients with stroke among 17,970 participants aged ≥ 45 years. Depressive symptoms were assessed using the 10-item Center for Epidemiologic Studies Depression (CES-D) Scale. We performed multivariable logistic regression models to estimate the associations between activities of daily life (ADL), instrumental activities of daily life (IADL) and pain with depressive symptoms.

**Results:**

Depressive symptoms were found among 40.2% of stroke patients, with a higher prevalence in females (48.2%) than males (32.7%). Prevalence of ADL limitations, IADL limitations and pain among stroke patients were 39.2, 49.8 and 14.0%, respectively. ADL and IADL limitations and pain were more prevalent among females and residents in rural areas. Multivariable regression analyses showed a significant association between ADL limitation (OR = 1.535, 95% CI = 1.168, 2.018), IADL limitation (OR = 1.666, 95% CI = 1.260, 2.203) and pain (OR = 2.122, 95% CI = 1.466, 3.073) with depressive symptoms. Stratified analyses revealed stronger associations among urban residents. Females had a higher association of ADL and IADL with depressive symptoms but similar in that of pain to the males. The impact of ADL and IADL in male patients is higher than in females, but the impact of pain on depressive symptoms is higher in female patients.

**Conclusion:**

Depressive symptoms are common amongst post-stroke patients in China and are significantly associated with functional disability and physical pain. Our findings have implications for practitioners on the early assessment of pain and depression after stroke. Future research should explore effective intervention measures for physical-mental stroke complications.

## Introduction

Stroke is a major cause of mortality, and has impact on long-term physical and cognitive impairment. According to the 2021 American Heart Association report on Heart Disease and Stroke Statistics, prevalence of stroke was estimated to be 2.7. This corresponds to ~7.6 million Americans aged 20 years and more who self-reported having a stroke and 874,000 stroke events (1). In China, the stroke burden has increased over the past three decades in both the rural and urban population, with 2.4 million new stroke events and 1.1 million stroke-related deaths annually (2). One study showed that the annual increase in functional disability more than tripled after stroke (3). Tang et al. assessed the cognition of 127 Swedish stroke survivors over 10 years and discovered that post-stroke cognitive impairment, whereby 46% of patients had a Mini-Mental State Examination (MMSE) score of <27 and 61% of patients had a Montreal Cognitive Assessment score of <25 (4).

Post-stroke depression (PSD) is also gaining recognition as a common condition. A meta-analysis of 61 cohorts including 25,488 patients reported that 31% of stroke patients developed depression at a point in time over 5 years (5). The literature from other counties shows that stroke patients who were female, with history of diabetes mellitus, higher national institutes of health stroke scale (NIHSS) score and lack of social support had a higher likelihood of developing PSD (6–10). The most consistent finding in the published literatures is that PSD is associated with stroke severity and the degree of cognitive impairment and physical function (such as, instrumental activities of daily living) (10). In addition, physical pain after stroke is a clinical issue that is both underdiagnosed and undertreated. It results in depression and cognitive troubles, and reduced quality of life (11). However, there is no data available regarding the epidemiology of chronic body pain and functional limitations, and its effect on the occurrence of depressive symptoms among patients with stroke in China. Research on the epidemiology of PSD and its risk factors is vital to develop prevention strategies, early detection, appropriate management and treatment.

This study aims to (1) examine the prevalence of depressive symptoms, functional limitations and physical pain among Chinese adults with stroke; (2) determine the associations between functional limitations and body pain with occurrence of depressive symptoms; (3) investigate gender and urban-rural disparities in these associations.

## Methods

### Data source

This study utilized data from the fourth wave of China Health and Retirement Longitudinal Study (CHARLS) 2018. There were 17,970 participants aged 45 years and older. Including 969 patients with stroke. The CHARLS is a population based nationally representative cohort study assessing health, economic and social aspects of aging in China. The data was collected *via* a survey in which four-stage, stratified, cluster sampling was used to randomly select eligible individuals (12). One hundred fifty counties in China were first selected with consideration to population size. Subsequently, three villages/communities were selected from each county as primary sampling units (PSUs). In each of the 450 PSUs, 80 households were randomly selected, with 24 for investigation. If the household had persons aged 45 years and over, one of them was randomly chosen, and both respondents and their spouses were interviewed using structured questionnaires.

Ethical approval for CHARLS was obtained from the Biomedical Ethics Review Committee of Peking University, and all interviewees were required to provide informed consent. The ethical approval number was IRB00001052–11015. A detailed description of the survey objectives and methods has been reported elsewhere (12).

### Measures

Depression was assessed by the 10-item Center for Epidemiologic Studies Depression Scale (CES-D 10) (13), which has been identified as a valid, reliable, and useful mental health assessment tool for those aged 60 and above in China (14). The responses included four options: (1) rarely (2) some days (1–2 days per week) (3) occasionally (3–4 days per week) (4) most of the time (5–7 days per week). The responses were recorded as 0 (rarely) to 3 (most of the time) for the negative questions in this study. For the two positive questions, the items were reversed as 3 (rarely) to 0 (most of the time). The total scores of the CESD-10 range from 0 to 30. In this study, a binary variable of mental health was constructed by defining an individual who's CES-D 10 score was <10 as having depressive symptoms.

Functional health was assessed by activities of daily living (ADL) and instrumental activities of daily living (IADL) limitations (15). The ADLs include six activities: bathing, dressing, feeding oneself, using the toilet, getting in or out of bed, and controlling urination and defecation. Answers were categorized as: "can do it by myself,” “have some difficulties,” “need help,” and “cannot do it.” The IADLs also include six activities: difficulty in doing household chores, cooking, shopping, making telephone calls, taking medications, and managing finances. Binary variables of ADL/ IADL were constructed, and ADL/ IADL disability was defined as having difficulty in one or more ADL/IADL items. This binary coding of ADL/ IADL variables was also used as dependent variables in the multivariable regression analysis.

The presence of chronic body pain was determined by two questions in this study. The participants were asked “In the past month, did you feel pain in your body?” If the response was “yes,” the participants were asked the following question, “On what part of your body do you feel pain? Please list all parts of your body (head, shoulder, arm, wrist, fingers, chest, stomach, back, waist, buttocks, leg, knees, ankles, toes, neck) where you are currently feeling pain. Each body part was recorded as a binary yes/no for having physical pain. This study considered a report of pain in any part of the body as “yes.”(16).

### Statistical analysis

We presented descriptive analysis of the prevalence of stroke, depressive symptoms, functional disability and body pain. The prevalence were weighted to account for the multi-stage PPS design of CHARLS. Multivariable logistic regression models were performed to estimate the association of functional disability (i.e., ADL, IADL) and body pain with depressive symptoms. Covariates included age, gender, marital status (married and partnered, unmarried, others), education (illiterate, primary and middle school, and high school and college), residence place (rural, urban), and chronic diseases (yes, no). Chronic diseases included hypertension, diabetes and heart diseases. We reported the adjusted Odds Ratio (OR) and 95% CIs. For sensitivity analyses, we performed multiple linear regressions with continuous depression/ADL/IADL measures and also included significantly difference variables as covariates. All the statistical analyses were conducted using STATA 15.0. *P* < 0.05 were considered statistically significant.

## Results

Overall, 969 stroke patients were identified from the dataset and analyzed in this study, with a median age of 66 years (range from 46 to 118 years) and 51.3% female. Most patients were married (78.5%) and resided in rural areas (62.2%). 24.7% of these Chinese patients who had a stroke were illiterate. Other demographic characteristics were presented in Table 1. Overall, 40.2% of stroke patients experienced depressive symptoms, with a higher prevalence in females than males (48.2 vs. 32.7%). Among Chinese stroke patients, the prevalence of ADL limitations, IADL limitations and chronic body pain is 39.2, 49.8 and 14.0%, respectively, with high proportions among female patients and rural residents (Table 2).

**Table 1:** Characteristics of the individuals with stroke in 2018.

**Variable**	**N**	**Unweighted %**	**Weighted %**
**Total**	969	100.0	100.0
**Age (year)**			
45–54	93	9.6	10.2
55–64	291	30.0	27.2
65–74	376	38.8	38.3
75 and above	209	21.6	24.4
**Gender**			
Male	478	49.3	51.3
Female	491	50.7	48.7
**Marital status**			
Married and partnered	212	21.9	21.5
Unmarried and other	757	78.1	78.5
**Education status**			
Illiterate	257	26.5	24.7
Primary and middle school	414	42.7	42.0
High school and college	298	30.8	33.3
**Residence place**			
Urban	299	30.9	37.8
Rural	670	69.1	62.2

**Table 2:** Co-occurring chronic conditions among individuals with stroke in China, by gender group (*n* = 969).

**Variable**	**Total**			**Males**			**Females**			***P*-value**
	***N***	**Unweighted**	**Weighted**	***N***	**Unweighted**	**Weighted**	***N***	**Unweighted**	**Weighted**	
		**%**	**%**		**%**	**%**		**%**	**%**	
**Total**	969	100.0	100.0	478	100.0	100.0	491	100.0	100.0	
**Age (year)**										
45–54	93	9.6	10.2	52	10.9	12.6	41	8.4	7.6	0.143
55–64	291	30	27.2	151	31.6	27.6	140	28.5	26.7	
65–74	376	38.8	38.3	169	35.4	36.8	207	42.2	39.8	
75 and above	209	21.6	24.4	106	22.2	22.9	103	21	25.9	
**Marital status**										
Married and partnered	212	21.9	21.5	67	14	11.1	145	29.5	32.5	<0.001
Unmarried and other	757	78.1	78.5	411	86	88.9	346	70.5	67.5	
**Education status**										
Illiterate	257	26.5	24.7	63	13.2	11.8	194	39.5	38.3	<0.001
Primary and middle school	414	42.7	42	209	43.7	44.3	205	41.8	39.6	
High school and college	298	30.8	33.3	206	43.1	43.9	92	18.7	22.1	
**Residence place**										
Urban	299	30.9	37.8	152	31.8	39.7	147	29.9	35.9	0.531
Rural	670	69.1	62.2	326	68.2	60.3	344	70.1	64.1	
**Depressive symptoms**										
No	561	57.9	59.8	308	64.4	67.3	253	51.5	51.8	<0.001
Yes	408	42.1	40.2	170	35.6	32.7	238	48.5	48.2	
**Hypertension**										
No	788	81.3	83.9	378	79.1	82.7	410	83.5	85.2	0.077
Yes	181	18.7	16.1	100	20.9	17.3	81	16.5	14.8	
**Diabetes**										
No	866	89.4	90.8	423	88.5	90.3	443	90.2	91.4	0.382
Yes	103	10.6	9.2	55	11.5	9.7	48	9.8	8.6	
**Heart diseases**										
No	842	86.9	89.6	419	87.7	90.5	423	86.2	88.6	0.487
Yes	127	13.1	10.4	59	12.3	9.5	68	13.9	11.4	
**ADL limitations**										
No	565	58.3	60.8	312	65.3	68.1	253	51.5	53.1	<0.001
Yes	404	41.7	39.2	166	34.7	31.9	238	48.5	46.9	
**IADL limitations**										
No	475	49.0	50.2	270	56.5	56.8	205	41.8	43.2	<0.001
Yes	494	51.0	49.8	208	43.5	43.2	286	58.3	56.8	
**Body pain**										
No	821	84.7	86.0	425	88.9	89.4	396	80.7	82.3	<0.001
Yes	148	15.3	14.0	53	11.1	10.6	95	19.4	17.7	

**Figure 1:**
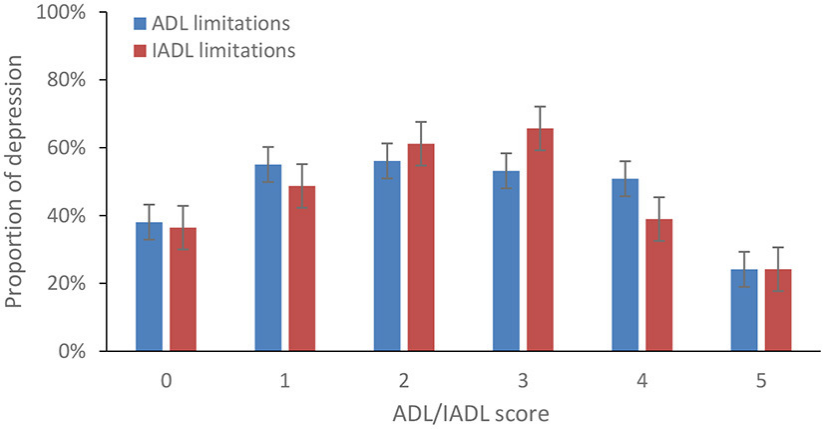
Percentage of depressive symptoms among patients with stroke by the score of functional limitations, 2018.

**Figure 2:**
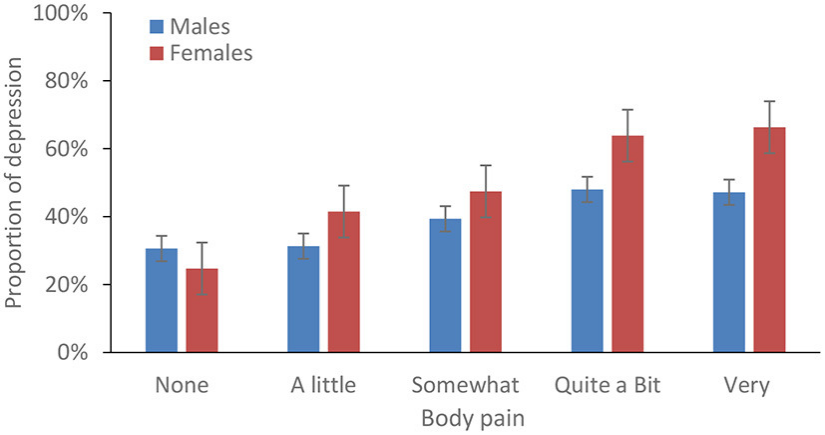
Percentage of depressive symptoms among patients with stroke by the degree of body pain and gender, 2018.

The proportion of patients with depressive symptoms increased and then decreased, as the ADL limitation score increased from 0 to 5. The results were similar for IADL limitations (Figure 1). The proportion of depressive symptoms increased as the body pain degree increased among female patients (Figure 2).

Amongst patients with stroke, depressive symptoms were associated with an increased likelihood of having ADL limitations (OR = 1.535, 95% CI = 1.168, 2.018), IADL limitations (OR = 1.666, 95% CI = 1.260, 2.203) and physical pain (OR = 2.122, 95% CI = 1.466, 3.073). The association of IADL limitations and chronic pain with depressive symptoms were statistically significant among stroke patients living in rural areas. However, the associations were not statistically significant among those in urban areas (Tables 3–5). Regarding sensitivity analyses, the results from to multiple linear regressions were consistent with our findings based on logistic regression models Supplementary Tables 1–6).

**Table 3:** Association of ADL limitations with depression among patients with stroke (*n* = 969).

**Variables**	**Total**				**Urban**				**Rural**			
	**OR^a^**	***P* value**	**95% CI**		**OR**	***P* value**	**95% CI**		**OR**	***P* value**	**95% CI**	
**ADL limitations (no)**	
Yes	1.535	0.002	1.168	2.018	1.745	0.036	1.038	2.932	1.470	0.020	1.062	2.036
**Age (45–54)**	
55-64	1.023	0.926	0.633	1.654	1.036	0.938	0.427	2.513	1.027	0.928	0.574	1.838
65-74	0.707	0.153	0.439	1.138	0.751	0.530	0.307	1.835	0.685	0.192	0.388	1.208
75 and above	0.509	0.014	0.297	0.872	0.683	0.440	0.260	1.796	0.436	0.013	0.226	0.839
**Gender (male)**	
Female	1.623	0.001	1.219	2.160	1.570	0.099	0.919	2.683	1.616	0.006	1.145	2.282
**Marital status (married)**	
Unmarried and other	1.040	0.822	0.739	1.463	1.078	0.813	0.580	2.004	0.994	0.975	0.656	1.504
**Level of education (illiterate)**	
Primary and middle school	1.343	0.087	0.958	1.882	1.367	0.431	0.628	2.972	1.313	0.161	0.897	1.922
High school and college	0.864	0.479	0.575	1.296	0.886	0.764	0.400	1.960	0.846	0.512	0.513	1.395
**Hypertension (no)**	
Yes	1.096	0.594	0.782	1.538	0.959	0.897	0.509	1.807	1.174	0.442	0.781	1.764
**Diabetes (no)**	
Yes	1.035	0.874	0.676	1.586	0.691	0.349	0.318	1.500	1.317	0.305	0.778	2.230
**Heart diseases (no)**	
Yes	1.051	0.803	0.710	1.555	0.844	0.626	0.426	1.670	1.220	0.429	0.746	1.995
**Residence place (urban)**	
Rural	1.249	0.148	0.924	1.687	-	-	-	-	-	-	-	-

Logistic regression models were used to examine the association of ADL limitations with depression.

OR^a^, the odds ratios estimated by adjusting for age, gender, marital status, level of education, hypertension, diabetes, heart diseases and residence place; ADL, activities of daily living; OR, odds ratio; CI, confidence interval.

**Table 4:** Association of IADL limitations with depression among patients with stroke.

**Variables**	**Total**				**Urban**				**Rural**			
	**OR^a^**	***P* value**	**95% CI**		**OR**	***P* value**	**95% CI**		**OR**	***P* value**	**95% CI**	
**ADL limitations (no)**	
Yes	1.666	<0.001	1.260	2.203	1.413	0.189	0.844	2.365	1.830	<0.001	1.306	2.566
**Age (45–54)**	
55-64	1.001	0.998	0.618	1.621	1.002	0.996	0.415	2.418	0.984	0.956	0.547	1.770
65-74	0.686	0.123	0.425	1.107	0.750	0.526	0.308	1.824	0.645	0.134	0.363	1.144
75 and above	0.480	0.008	0.279	0.826	0.707	0.479	0.271	1.846	0.381	0.005	0.196	0.744
**Gender (male)**	
Female	1.618	0.001	1.215	2.156	1.678	0.056	0.988	2.849	1.559	0.012	1.101	2.206
**Marital status (married)**	
Unmarried and other	1.022	0.902	0.726	1.438	1.103	0.756	0.595	2.046	0.968	0.877	0.639	1.467
**Level of education (illiterate)**	
Primary and middle school	1.406	0.050	1.000	1.976	1.371	0.427	0.630	2.983	1.389	0.095	0.945	2.041
High school and college	0.923	0.701	0.612	1.391	0.871	0.736	0.391	1.942	0.907	0.704	0.548	1.502
**Hypertension (no)**	
Yes	1.040	0.820	0.740	1.462	0.938	0.844	0.497	1.771	1.114	0.606	0.739	1.680
**Diabetes (no)**	
Yes	1.025	0.910	0.668	1.572	0.690	0.347	0.318	1.495	1.314	0.311	0.774	2.231
**Heart diseases (no)**	
Yes	1.093	0.657	0.738	1.617	0.854	0.650	0.433	1.687	1.292	0.311	0.788	2.119
**Residence place (urban)**	
Rural	1.215	0.206	0.899	1.644	-	-	-	-	-	-	-	-

Logistic regression models were used to examine the association of IADL limitations with depression.

OR^a^, the odds ratios estimated by adjusting for age, gender, marital status, level of education, and residence place; IADL, instrumental activities of daily living; OR, odds ratio; CI, confidence interval.

**Table 5:** Association of body pain with depression among patients with stroke.

**Variables**	**Total**				**Urban**				**Rural**			
	**OR^a^**	***P* value**	**95% CI**		**OR**	***P* value**	**95% CI**		**OR**	***P* value**	**95% CI**	
**Body pain (no)**	
Yes	2.122	<0.001	1.466	3.073	1.586	0.244	0.730	3.448	2.394	<0.001	1.557	3.681
**Age (45–54)**	
55-64	1.035	0.889	0.639	1.677	0.968	0.943	0.402	2.331	1.051	0.869	0.584	1.889
65-74	0.743	0.224	0.461	1.199	0.789	0.600	0.325	1.917	0.709	0.239	0.400	1.256
75 and above	0.571	0.041	0.334	0.976	0.785	0.621	0.301	2.048	0.465	0.022	0.241	0.896
**Gender (male)**	
Female	1.643	0.001	1.234	2.187	1.665	0.059	0.981	2.825	1.626	0.006	1.149	2.300
**Marital status (married)**	
Unmarried and other	1.016	0.929	0.721	1.430	1.090	0.784	0.588	2.023	0.965	0.867	0.636	1.465
**Level of education (illiterate)**	
Primary and middle school	1.386	0.060	0.986	1.948	1.349	0.450	0.621	2.928	1.356	0.122	0.922	1.994
High school and college	0.932	0.737	0.618	1.406	0.821	0.623	0.374	1.802	0.975	0.923	0.587	1.621
**Hypertension (no)**	
Yes	1.098	0.589	0.781	1.544	0.995	0.988	0.530	1.869	1.172	0.450	0.776	1.771
**Diabetes (no)**	
Yes	1.045	0.841	0.681	1.604	0.724	0.415	0.333	1.574	1.295	0.342	0.760	2.205
**Heart diseases (no)**	
Yes	1.037	0.856	0.700	1.536	0.830	0.592	0.420	1.641	1.196	0.479	0.728	1.963
**Residence place (urban)**	
Rural	1.238	0.166	0.915	1.674	-	-	-	-	-	-	-	-

Logistic regression models were used to examine the association of body pain with depression.

OR^a^, the odds ratios estimated by adjusting for age, gender, marital status, level of education, and residence place; OR, odds ratio; CI, confidence interval.

**Figure 3:**
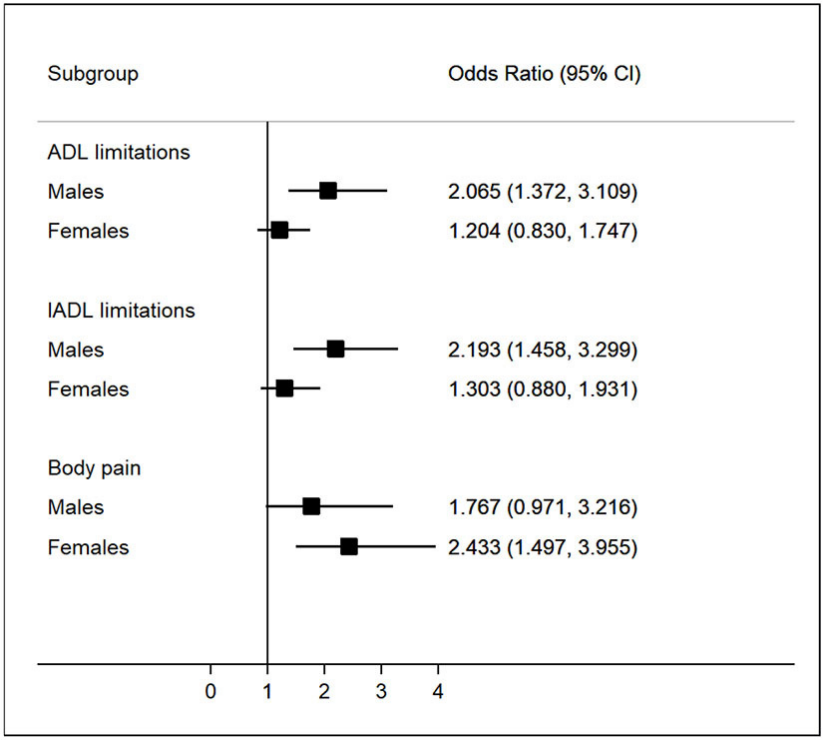
Association of function limitations and body pain with depression among patients with stroke stratified by gender.

Figure 3 reveals the gender differences in the associations between depressive symptoms and functional difficulties and body pain among patients with stroke. We found a stronger association between ADL/IADL limitations and depressive symptoms among males, compared to females with stroke. In contrast, there was a stronger association between physical pain and depressive symptoms among females, compared to males.

## Discussion

### Principal findings

We demonstrated that 40.2% of stroke survivors had depressive symptoms among participants in CHARLS 2018. Depressive symptoms were more prevalent in females. Among patients who had a stroke event, the prevalence of ADL limitations, IADL limitations and physical pain was 39.2, 49.8 and 14.0%, respectively. Females and rural residents had a higher prevalence of ADL/IADL limitations and pain. Importantly, our study revealed that depressive symptoms was associated with lower functional status and increased physical pain. These relationships were greater in urban areas than in rural areas. The association between ADL and IADL limitations and depressive symptoms were stronger in males. However, physical pain had a greater association with depressive symptoms in females. Our results have implications for clinicians on the evaluation and treatment of patients with post-stroke depressive symptoms, especially rural area doctors. Early intervention of depressive symptoms will likely prevent the trajectory toward clinical depressive symptoms and other mental health conditions.

### Literature comparisons

We acknowledge that this study assessed depressive symptoms, instead of diagnosed clinical depressive symptoms using ICD codes. This may have limited the ability to make comparisons with the literature that examined clinically diagnosed depressive symptoms. The prevalence of depressive symptoms among patients with stroke (40.2%) reported in this study is higher than published literature in China (17, 18) and other countries (6, 19). Wang et al. (18) found that the incidence of PSD was 25.4, 17.6 and 12.4%, at 2 weeks, 3 and 12 months after stroke, respectively. There are differences in PSD prevalence may be due to the neglect of PSD classification according to the different phase after stroke. The overall PSD incidence was 12.8% in Sweden and Finland (19) and 33% in Australia (6) may be explained by that physicians' awareness of PSD. These countries have greater mental health awareness and healthcare systems that have preventive and treatment measures for depressive and anxiety symptoms (19).

In addition, our findings on females having a higher prevalence of depressive symptoms among patients with stroke is consistent with published articles. Medeiros et al. (20) reviewed the literature on PSD and revealed that PSD was more common in females than males (OR 1.33–2.40 across studies) (20). The cohort study by Wang et al. in mainland China showed that sex was an independent risk factor for PSD in the acute stage of stroke (18). Females may be more open and less stigmatized in self-reporting depressive symptoms and poorer mental health, compared to males. Also, it is possible that females are more vulnerable to psychological stress and loss of function and social connection following a stroke (8, 21–24).

Our findings on the positive association between reduced functional status and depressive symptoms are consistent with the literature. The systematic reviews by Hackett and Pickles and Johnson et al. reported that the severity of disability was significantly related to PSD in 16 of 18 studies (5) and 24 of 30 studies (25), respectively. In contrast to our study, current literature suggests that the strength of the correlation between PSD and impairment in ADLs is relatively weak, explaining only about 10% of the variance of severity of PSD (26). To better understand the underlying mechanism of how functional status impacts depressive symptoms, larger prospective studies in future research may be useful.

We found a greater association between ADL/IADL limitations and depressive symptoms in males who had a stroke. Studies have also revealed that males had the same or higher likelihood of PSD compared to females (27–29). Males, especially those in Asia, are more affected by the loss of work productivity and income from having a stroke and functional disability.

Our findings on the prevalence of physical pain (14%) among Chinese stroke patients is within reported prevalence of post-stroke pain that range from 10.6 to 55.3% (30). The wide range of prevalence is likely a reflection of differences in definition of pain, parts of the body studied, study design and target population. Pain after stroke is often not reported by patients unless they are explicitly asked by medical staff (31). Even when identified, pain after stroke may not be sufficiently treated. In one retrospective study, it was found that two-thirds of patients with pain had inadequate pain treatment or were not prescribed any treatment (32). Our study revealed experiencing pain was associated with depressive symptoms. A population-based study in Sweden also showed that pain after stroke had a strong relationship with depression (33). We found that female stroke patients had a higher prevalence of chronic body pain. Harrison et al. also revealed that sex was a risk factor for the development of post stroke pain (30). This could be the first study to report this finding. Females may be more susceptible to hormonal changes after stroke and sensitive to somatosensory effects including pain. We propose for future research to examine the gender differences in this association further.

Several studies support our findings on rural stroke patients having more neurological deficits and chronic body pain (30, 34). This was mainly due to the lack of medical services including medical insurance, medicine, physician and medical equipment, compared to urban residents (30, 34). This study found that the associations between IADL limitations and chronic pain with depressive symptoms were statistically significant among stroke patients in rural areas. The burden of stroke burden in China has increased over the past 30 years and remains particularly high in rural areas (2). There are disparities in the healthcare systems and infrastructures between the urban and rural parts of China, (35) and those in rural areas have less access to medical services (36). Current evidence shows that there exists an urban-rural gap in service quality and accessibility in the field of stroke rehabilitation and care (37), and our findings support this. Furthermore, several studies from other counties show that age, marital status, education, sleep duration, interpersonal relationships, activity participation and other demographic and clinical factors are also associated with depressive symptoms after stroke (38–43).

### Clinical and public health implications

The incidence of stroke has remained high in China and most developed countries (1, 2). Practitioners have primarily focused on the preventive and treatment strategies for reducing physical functional impairment of stroke patients. However, pain and depression after stroke, which have significant influence on the quality of daily life of patients, are still underdiagnosed and undertreated (11). A recommendation for practice is for doctors and allied health staff to assess pain and depressive symptoms of stroke patients at the outpatient clinics and hospitals. It is important for healthcare staff to recognize early signs of physical pain and depressive symptoms, in order to apply timely intervention measures to prevent the trajectory toward clinical depression and poor mental health.

Additionally, our study demonstrated that stroke patients who were female and rural resident had a higher prevalence of functional disability and physical pain. Hence, we suggest that practitioners in China and other countries could provide added intervention measures for post-stroke pain and depressive symptoms for females and rural residents. Another recommendation could be to for more in-depth assessment of male stroke patients who may be less likely to self-report depressive symptoms. From a public health perspective, narrowing the gap between the rural-urban divide in healthcare accessibility and quality in the field of stroke rehabilitation and care may improve functional status, physical pain and mental health of stroke patients in China.

### Strengths and limitations

A key strength of this study is the use of a nationally representative dataset to collect comprehensive data on reliable estimates on depressive symptoms, functional disability, and physical pain in different parts of the body among stroke survivors. However, our findings should be interpreted within the context of some limitations. First, the cross-sectional study design precluded establishing causal relationships between socio-demographic characteristics and risk factors with depressive symptom among patients with stroke. Our findings warrant further investigation by examining the changes of risk factors over time using time series or longitudinal data. Second, we acknowledge that this study assessed depressive symptoms by using CES-D 10, instead of diagnosed clinical depression using ICD codes. This may have limited the ability to make comparisons with the literature that examined clinically diagnosed depression. However, our study still revealed important associations between post-stroke depressive symptoms with functional status and physical pain. It is important for interventions to target mental health at an earlier trajectory. Third, functional limitations and general body pain (unspecified cause of pain) were self-reported. There may be recall bias and social acceptability bias. These biases likely led to under-reporting with results biased toward the null. However, our findings still have value and important implications. Lastly, this study only include young adult Chinese due to lack of available data in CHARLS, which may limit generalisability to younger adults.

In conclusion, depressive symptoms is common among post-stroke patients in China and is significantly associated with functional disability and physical pain across different parts of the body. Our findings have implications to practitioners on the early assessment of pain and depression after stroke. Future research should explore effective intervention measures for physical-mental stroke complications.

## Data availability statement

The original contributions presented in the study are included in the article/Supplementary material, further inquiries can be directed to the corresponding author.

## Ethics statement

The Biomedical Ethics Review Committee of Peking University approved the China Health and Retirement Longitudinal study, and all interviewees were required to provide written informed consent. The ethical approval number was IRB00001052-11015.

## Author contributions

WZ and CA conceived and designed the study. WZ, YW, and LS did the initial analysis and supervised data analysis. WZ, LuZ, and YW wrote the first draft of the paper. GS, AK, XC, YJ, LiZ, CA, and LS critically revised the first draft. All authors reviewed and approved the final version of the paper submitted for publication.

## Conflict of interest

The authors declare that the research was conducted in the absence of any commercial or financial relationships that could be construed as a potential conflict of interest.

## Publisher's note

All claims expressed in this article are solely those of the authors and do not necessarily represent those of their affiliated organizations, or those of the publisher, the editors and the reviewers. Any product that may be evaluated in this article, or claim that may be made by its manufacturer, is not guaranteed or endorsed by the publisher.
