# Supplementary material for: The association between functional status and physical pain with depressive symptoms after a stroke event: A cross-sectional analysis of the China Health and Retirement Longitudinal Study 2018
Source: Front Psychiatry. 2022 Sep 12;13:927856. doi: 10.3389/fpsyt.2022.927856 (PMC9512144; doi:10.3389/fpsyt.2022.927856)
Supplement: Supplementary file 1 [file Table_1.docx]

**Supplemental tables**

**Table S1.** Association of ADL limitations with depression among patients with stroke, by residence place (linear regressions)

| **Variables** | **Total** | | | | **Urban** | | | | **Rural** | | | |
| --- | --- | --- | --- | --- | --- | --- | --- | --- | --- | --- | --- | --- |
|  | Coef. | P value | 95% CI | | Coef. | P value | 95% CI | | Coef. | P value | 95% CI | |
| ADL limitations | 1.428 | <0.001 | 1.091 | 1.765 | 1.735 | <0.001 | 1.058 | 2.412 | 1.353 | <0.001 | 0.957 | 1.749 |
| Age (45-54) |  |  |  |  |  |  |  |  |  |  |  |  |
| 55-64 | -0.456 | 0.578 | -2.064 | 1.152 | 0.600 | 0.672 | -2.189 | 3.388 | -0.936 | 0.353 | -2.913 | 1.042 |
| 65-74 | -1.926 | 0.018 | -3.525 | -0.327 | -0.824 | 0.564 | -3.635 | 1.987 | -2.419 | 0.015 | -4.363 | -0.475 |
| 75 and above | -2.056 | 0.029 | -3.895 | -0.216 | 0.222 | 0.889 | -2.900 | 3.344 | -3.042 | 0.009 | -5.326 | -0.758 |
| Gender (male) |  |  |  |  |  |  |  |  |  |  |  |  |
| Female | 2.038 | <0.001 | 1.061 | 3.015 | 0.431 | 0.617 | -1.265 | 2.127 | 2.757 | <0.001 | 1.544 | 3.970 |
| Marital status (married) |  |  |  |  |  |  |  |  |  |  |  |  |
| Unmarried and other | -1.049 | 0.088 | -2.255 | 0.158 | -1.527 | 0.143 | -3.576 | 0.522 | -1.008 | 0.186 | -2.504 | 0.488 |
| Level of education (illiterate) |  |  |  |  |  |  |  |  |  |  |  |  |
| Primary and middle school | 0.141 | 0.815 | -1.038 | 1.319 | -0.707 | 0.601 | -3.370 | 1.955 | 0.377 | 0.581 | -0.966 | 1.721 |
| High school and college | -1.527 | 0.032 | -2.925 | -0.129 | -2.969 | 0.028 | -5.606 | -0.331 | -0.714 | 0.423 | -2.465 | 1.036 |
| Hypertension (no) |  |  |  |  |  |  |  |  |  |  |  |  |
| Yes | 0.773 | 0.199 | -0.408 | 1.953 | 0.333 | 0.760 | -1.816 | 2.483 | 0.915 | 0.209 | -0.514 | 2.344 |
| Diabetes (no) |  |  |  |  |  |  |  |  |  |  |  |  |
| Yes | 0.461 | 0.536 | -1.001 | 1.923 | -0.559 | 0.650 | -2.985 | 1.867 | 0.979 | 0.294 | -0.853 | 2.811 |
| Heart diseases (no) |  |  |  |  |  |  |  |  |  |  |  |  |
| Yes | 0.293 | 0.665 | -1.035 | 1.621 | -0.578 | 0.618 | -2.856 | 1.700 | 0.782 | 0.355 | -0.879 | 2.442 |
| Residence place (urban) |  |  |  |  |  |  |  |  |  |  |  |  |
| Rural | 0.973 | 0.065 | -0.059 | 2.005 | - | - | - | - | - | - | - | - |

**Notes:** Multiple linear regression models were used to examine the association of ADL limitations with depression. Coefficients estimated by adjusting for age, gender, marital status, level of education, hypertension, diabetes, heart diseases and residence place, ADL, activities of daily living; CI, confidence interval.

**Table S2.** Association of IADL limitations with depression among patients with stroke, by residence place (linear regressions)

| **Variables** | **Total** | | | | **Urban** | | | | **Rural** | | | |
| --- | --- | --- | --- | --- | --- | --- | --- | --- | --- | --- | --- | --- |
|  | Coef. | P value | 95% CI | | Coef. | P value | 95% CI | | Coef. | P value | 95% CI | |
| IADL limitations | 1.570 | <0.001 | 1.240 | 1.900 | 2.261 | <0.001 | 1.589 | 2.932 | 1.402 | <0.001 | 1.017 | 1.788 |
| Age (45-54) |  |  |  |  |  |  |  |  |  |  |  |  |
| 55-64 | -0.528 | 0.515 | -2.120 | 1.063 | 0.113 | 0.934 | -2.578 | 2.804 | -0.834 | 0.405 | -2.802 | 1.134 |
| 65-74 | -2.060 | 0.011 | -3.643 | -0.476 | -1.278 | 0.356 | -3.999 | 1.443 | -2.471 | 0.012 | -4.406 | -0.536 |
| 75 and above | -2.520 | 0.007 | -4.350 | -0.690 | -0.160 | 0.917 | -3.178 | 2.858 | -3.491 | 0.003 | -5.781 | -1.202 |
| Gender (male) |  |  |  |  |  |  |  |  |  |  |  |  |
| Female | 2.046 | <0.001 | 1.079 | 3.012 | 0.826 | 0.319 | -0.804 | 2.456 | 2.648 | <0.001 | 1.439 | 3.857 |
| Marital status (married) |  |  |  |  |  |  |  |  |  |  |  |  |
| Unmarried and other | -1.483 | 0.015 | -2.679 | -0.288 | -1.634 | 0.105 | -3.614 | 0.346 | -1.570 | 0.039 | -3.060 | -0.080 |
| Level of education (illiterate) |  |  |  |  |  |  |  |  |  |  |  |  |
| Primary and middle school | 0.497 | 0.405 | -0.674 | 1.668 | 0.084 | 0.949 | -2.506 | 2.674 | 0.645 | 0.345 | -0.696 | 1.985 |
| High school and college | -0.952 | 0.181 | -2.348 | 0.444 | -1.614 | 0.225 | -4.229 | 1.002 | -0.360 | 0.686 | -2.107 | 1.387 |
| Hypertension (no) |  |  |  |  |  |  |  |  |  |  |  |  |
| Yes | 0.655 | 0.272 | -0.514 | 1.824 | 0.161 | 0.879 | -1.916 | 2.238 | 0.839 | 0.247 | -0.584 | 2.262 |
| Diabetes (no) |  |  |  |  |  |  |  |  |  |  |  |  |
| Yes | 0.486 | 0.510 | -0.960 | 1.933 | -0.367 | 0.758 | -2.710 | 1.975 | 0.969 | 0.297 | -0.854 | 2.791 |
| Heart diseases (no) |  |  |  |  |  |  |  |  |  |  |  |  |
| Yes | 0.507 | 0.449 | -0.807 | 1.820 | -0.714 | 0.523 | -2.911 | 1.484 | 1.154 | 0.170 | -0.494 | 2.801 |
| Residence place (urban) |  |  |  |  |  |  |  |  |  |  |  |  |
| Rural | 0.863 | 0.098 | -0.160 | 1.886 | - | - | - | - | - | - | - | - |

**Notes:** Multiple linear regression models were used to examine the association of IADL limitations with depression. Coefficients estimated by adjusting for age, gender, marital status, level of education, and residence place. IADL, instrumental activities of daily living; CI, confidence interval.

**Table S3.** Association of body pain with depression among patients with stroke, by residence place (linear regressions)

| **Variables** | **Total** | | | | **Urban** | | | | **Rural** | | | |
| --- | --- | --- | --- | --- | --- | --- | --- | --- | --- | --- | --- | --- |
|  | Coef. | P value | 95% CI | | Coef. | P value | 95% CI | | Coef. | P value | 95% CI | |
| Body pain (no) |  |  |  |  |  |  |  |  |  |  |  |  |
| Yes | 3.151 | <0.001 | 1.840 | 4.462 | 1.999 | 0.166 | -0.834 | 4.833 | 3.600 | <0.001 | 2.103 | 5.098 |
| Age (45-54) |  |  |  |  |  |  |  |  |  |  |  |  |
| 55-64 | -0.377 | 0.654 | -2.031 | 1.276 | 0.244 | 0.870 | -2.679 | 3.166 | -0.718 | 0.485 | -2.734 | 1.298 |
| 65-74 | -1.645 | 0.050 | -3.288 | -0.002 | -0.482 | 0.748 | -3.433 | 2.469 | -2.169 | 0.032 | -4.150 | -0.188 |
| 75 and above | -1.277 | 0.183 | -3.160 | 0.606 | 0.506 | 0.762 | -2.785 | 3.796 | -2.114 | 0.072 | -4.422 | 0.193 |
| Gender (male) |  |  |  |  |  |  |  |  |  |  |  |  |
| Female | 2.100 | <0.001 | 1.094 | 3.107 | 0.766 | 0.397 | -1.011 | 2.543 | 2.792 | <0.001 | 1.553 | 4.031 |
| Marital status (married) |  |  |  |  |  |  |  |  |  |  |  |  |
| Unmarried and other | -1.177 | 0.063 | -2.417 | 0.063 | -1.500 | 0.171 | -3.653 | 0.654 | -1.158 | 0.136 | -2.682 | 0.366 |
| Level of education (illiterate) |  |  |  |  |  |  |  |  |  |  |  |  |
| Primary and middle school | 0.162 | 0.793 | -1.052 | 1.376 | -0.789 | 0.581 | -3.604 | 2.026 | 0.408 | 0.559 | -0.963 | 1.779 |
| High school and college | -1.498 | 0.042 | -2.944 | -0.051 | -3.658 | 0.010 | -6.434 | -0.882 | -0.351 | 0.701 | -2.148 | 1.446 |
| Hypertension (no) |  |  |  |  |  |  |  |  |  |  |  |  |
| Yes | 0.907 | 0.143 | -0.306 | 2.120 | 0.847 | 0.458 | -1.400 | 3.094 | 0.981 | 0.186 | -0.476 | 2.438 |
| Diabetes (no) |  |  |  |  |  |  |  |  |  |  |  |  |
| Yes | 0.345 | 0.652 | -1.157 | 1.847 | -0.135 | 0.918 | -2.705 | 2.435 | 0.545 | 0.566 | -1.320 | 2.410 |
| Heart diseases (no) |  |  |  |  |  |  |  |  |  |  |  |  |
| Yes | 0.415 | 0.551 | -0.950 | 1.780 | -1.012 | 0.405 | -3.404 | 1.380 | 1.094 | 0.204 | -0.594 | 2.783 |
| Residence place (urban) |  |  |  |  |  |  |  |  |  |  |  |  |
| Rural | 1.149 | 0.034 | 0.089 | 2.209 | - | - | - | - | - | - | - | - |

**Notes:** Multiple linear regression models were used to examine the association of body pain with depression. Coefficients estimated by adjusting for age, gender, marital status, level of education, and residence place. CI, confidence interval.

**Table S4.** Association of ADL limitations with depression among patients with stroke, by residence place (linear regressions including variables with significant difference)

| **Variables** | **Total** | | | | **Urban** | | | | **Rural** | | | |
| --- | --- | --- | --- | --- | --- | --- | --- | --- | --- | --- | --- | --- |
|  | Coef. | P value | 95% CI | | Coef. | P value | 95% CI | | Coef. | P value | 95% CI | |
| ADL limitations | 1.462 | <0.001 | 1.126 | 1.799 | 1.738 | <0.001 | 1.065 | 2.410 | 1.371 | <0.001 | 0.977 | 1.765 |
| Age (45-54) |  |  |  |  |  |  |  |  |  |  |  |  |
| 55-64 | -0.493 | 0.547 | -2.099 | 1.113 | 0.626 | 0.656 | -2.139 | 3.392 | -1.049 | 0.297 | -3.022 | 0.923 |
| 65-74 | -1.961 | 0.016 | -3.552 | -0.370 | -0.731 | 0.604 | -3.506 | 2.044 | -2.412 | 0.015 | -4.348 | -0.476 |
| 75 and above | -1.952 | 0.034 | -3.752 | -0.152 | 0.682 | 0.658 | -2.348 | 3.713 | -2.899 | 0.011 | -5.137 | -0.661 |
| Gender (male) |  |  |  |  |  |  |  |  |  |  |  |  |
| Female | 2.017 | <0.001 | 1.058 | 2.976 | 0.708 | 0.387 | -0.901 | 2.318 | 2.829 | <0.001 | 1.622 | 4.036 |
| Level of education (illiterate) |  |  |  |  |  |  |  |  |  |  |  |  |
| Primary and middle school | -0.047 | 0.937 | -1.219 | 1.124 | -0.851 | 0.524 | -3.477 | 1.774 | 0.269 | 0.694 | -1.072 | 1.611 |
| High school and college | -1.958 | 0.004 | -3.294 | -0.621 | -2.970 | 0.027 | -5.592 | -0.348 | -0.808 | 0.365 | -2.558 | 0.942 |

**Notes:** Multiple linear regression models were used to examine the association of ADL limitations with depression. Coefficients estimated by adjusting for age, gender, and level of education. ADL, activities of daily living; CI, confidence interval.

**Table S5.** Association of IADL limitations with depression among patients with stroke, by residence place (linear regressions including variables with significant difference)

| **Variables** | **Total** | | | | **Urban** | | | | **Rural** | | | |
| --- | --- | --- | --- | --- | --- | --- | --- | --- | --- | --- | --- | --- |
|  | Coef. | P value | 95% CI | | Coef. | P value | 95% CI | | Coef. | P value | 95% CI | |
| IADL limitations | 1.598 | <0.001 | 1.270 | 1.927 | 2.266 | <0.001 | 1.601 | 2.932 | 1.403 | <0.001 | 1.017 | 1.788 |
| Age (45-54) |  |  |  |  |  |  |  |  |  |  |  |  |
| 55-64 | -0.570 | 0.481 | -2.157 | 1.018 | 0.079 | 0.953 | -2.584 | 2.743 | -0.943 | 0.346 | -2.907 | 1.020 |
| 65-74 | -2.171 | 0.007 | -3.748 | -0.595 | -1.298 | 0.342 | -3.983 | 1.386 | -2.540 | 0.010 | -4.471 | -0.609 |
| 75 and above | -2.720 | 0.003 | -4.542 | -0.898 | -0.147 | 0.923 | -3.126 | 2.832 | -3.647 | 0.002 | -5.932 | -1.362 |
| Gender (male) |  |  |  |  |  |  |  |  |  |  |  |  |
| Female | 1.917 | <0.001 | 0.958 | 2.875 | 0.834 | 0.301 | -0.752 | 2.420 | 2.640 | <0.001 | 1.429 | 3.850 |
| Marital status (married) |  |  |  |  |  |  |  |  |  |  |  |  |
| Unmarried and other | -1.476 | 0.015 | -2.670 | -0.282 | -1.640 | 0.101 | -3.601 | 0.320 | -1.531 | 0.044 | -3.020 | -0.042 |
| Level of education (illiterate) |  |  |  |  |  |  |  |  |  |  |  |  |
| Primary and middle school | 0.369 | 0.534 | -0.795 | 1.533 | -0.041 | 0.975 | -2.590 | 2.509 | 0.568 | 0.406 | -0.772 | 1.908 |
| High school and college | -1.336 | 0.050 | -2.674 | 0.001 | -1.652 | 0.211 | -4.248 | 0.944 | -0.450 | 0.613 | -2.196 | 1.297 |

**Notes:** Multiple linear regression models were used to examine the association of IADL limitations with depression. Coefficients estimated by adjusting for age, gender, marital status, and level of education. IADL, instrumental activities of daily living; CI, confidence interval.

**Table S6.** Association of body pain with depression among patients with stroke, by residence place (linear regressions including variables with significant difference)

| **Variables** | **Total** | | | | **Urban** | | | | **Rural** | | | |
| --- | --- | --- | --- | --- | --- | --- | --- | --- | --- | --- | --- | --- |
|  | Coef. | P value | 95% CI | | Coef. | P value | 95% CI | | Coef. | P value | 95% CI | |
| Body pain (no) |  |  |  |  |  |  |  |  |  |  |  |  |
| Yes | 3.165 | <0.001 | 1.853 | 4.477 | 1.798 | 0.205 | -0.990 | 4.585 | 3.675 | <0.001 | 2.176 | 5.174 |
| Age (45-54) |  |  |  |  |  |  |  |  |  |  |  |  |
| 55-64 | -0.457 | 0.587 | -2.107 | 1.194 | 0.240 | 0.871 | -2.660 | 3.140 | -0.875 | 0.393 | -2.887 | 1.137 |
| 65-74 | -1.648 | 0.048 | -3.283 | -0.013 | -0.463 | 0.755 | -3.380 | 2.454 | -2.179 | 0.031 | -4.152 | -0.205 |
| 75 and above | -1.053 | 0.262 | -2.897 | 0.790 | 0.844 | 0.604 | -2.354 | 4.042 | -1.957 | 0.090 | -4.218 | 0.304 |
| Gender (male) |  |  |  |  |  |  |  |  |  |  |  |  |
| Female | 2.186 | <0.001 | 1.193 | 3.179 | 0.957 | 0.266 | -0.735 | 2.648 | 2.879 | <0.001 | 1.647 | 4.112 |
| Level of education (illiterate) |  |  |  |  |  |  |  |  |  |  |  |  |
| Primary and middle school | 0.087 | 0.888 | -1.127 | 1.301 | -1.050 | 0.457 | -3.823 | 1.724 | 0.298 | 0.669 | -1.071 | 1.668 |
| High school and college | -1.576 | 0.033 | -3.021 | -0.130 | -3.762 | 0.008 | -6.518 | -1.006 | -0.444 | 0.628 | -2.241 | 1.354 |
| Residence place (urban) |  |  |  |  |  |  |  |  |  |  |  |  |
| Rural | 1.090 | 0.044 | 0.030 | 2.149 | - | - | - | - | - | - | - | - |

**Notes:** Multiple linear regression models were used to examine the association of body pain with depression. Coefficients estimated by adjusting for age, gender, level of education, and residence place. CI, confidence interval.
